# Accidental swallowing of partial denture: a case report

**DOI:** 10.1186/1757-1626-2-9363

**Published:** 2009-12-21

**Authors:** Yusuf Tanrikulu, Serap Erel, Kemal Kismet, Mefaret Sahin, Evren K Ortac, Mehmet A Akkus

**Affiliations:** 1 Ankara Training and Research Hospital, Ulucanlar, Ankara, 06340, Turkey

## Abstract

We describe a 42-year-old age woman who accidentally swallowed her lower denture, which was composed of eleven teeth. The daily descent of the denture was followed by plain abdominal radiography and physical examination. The image was localized at the left upper quadrant on admission day, but it stopped on its way at the right lower quadrant on day two and three. Since the patient's complaints increased we planned surgical removal of the denture. In this report, we had discussed the diagnosis, follow up and treatment options of swallowed partial denture with current literature review.

## Introduction

Foreign body aspiration is a common pediatric problem that commonly affects children of all ages and has been a recognized cause of accidental deaths. In adults aspiration of teeth and dental restorations is a recognized, yet an infrequent happening in the literature. Main reasons of aspiration are maxillofacial trauma, dental treatment procedures or ethanol intoxication and dementia [1-3].

The majority of foreign bodies entering the oropharynx will pass into the alimentary canal and pass without incidence, though there is a danger of perforation of the gut which can have very serious consequences including death.

If the material used in the construction of denture is methylmethacrylate, which is radiolucent, early diagnosis of an impacted or ingested denture in many cases is complicated. Herein, we present surgical treatment of accidentally swallowed partial denture composing of eleven units in a 42-year-old woman.

## Case presentation

A 42-year-old Turkish woman with a history of swallowing of lower partial denture while eating her meal 4 hours ago presented to emergency department with complaints of abdominal pain and nausea. On physical examination her vital signs were stable and there was just abdominal tenderness.

**Figure 1:**
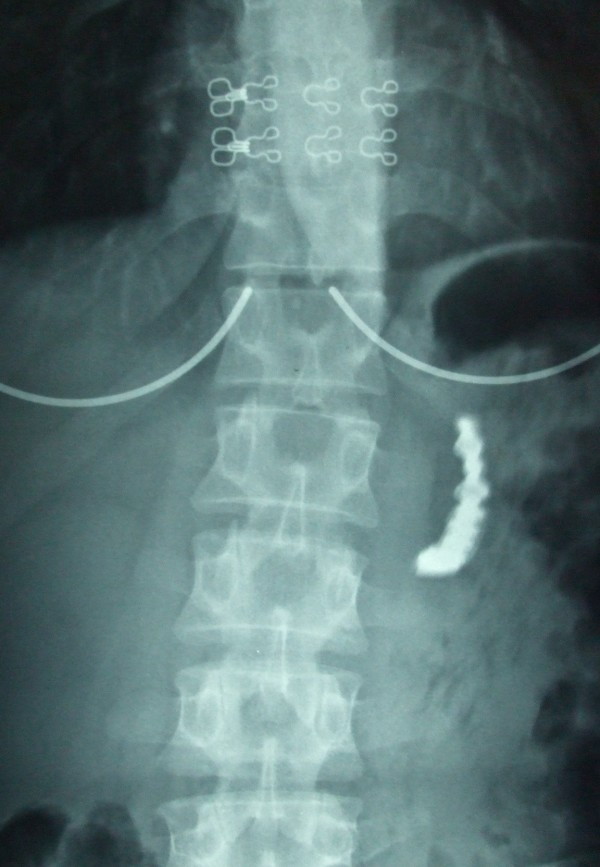
**Image of partial denture at left upper quadrant of the plain abdominal radiograph on admission day**.

**Figure 2:**
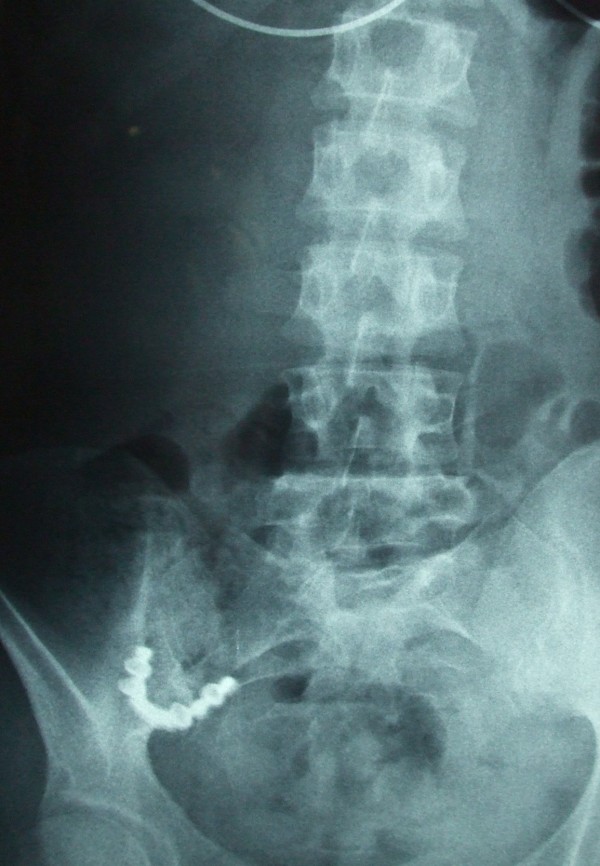
**Image of partial denture at right lower quadrant**.

**Figure 3:**
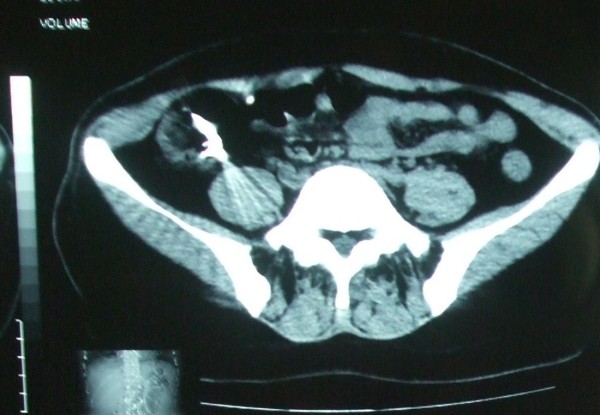
**Image of partial denture at ileocecal region at lower computurizedc tomography**.

Complete blood count and blood chemistry were normal. Plane abdominal radiograph of the patient on left upper quadrant, revealed foreign body in accordance with denture (Figure 1). On follow up radiograph at second day, the image of prosthesis was displaced to right lower quadrant (Figure 2). On third day, the patient started complaining about increased abdominal pain, nausea, vomiting and abdominal distention. Control abdominal radiograph demonstrated persistence of denture on the same location. Computerized tomography revealed the denture to be lodged in the iliocecal region (Figure 3).

**Figure 4:**
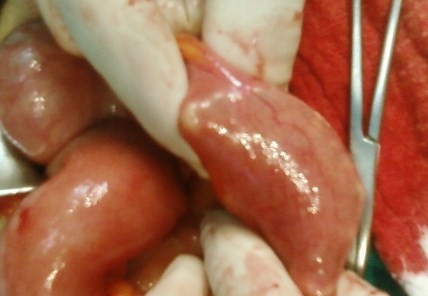
**Partial denture at ileum**.

**Figure 5:**
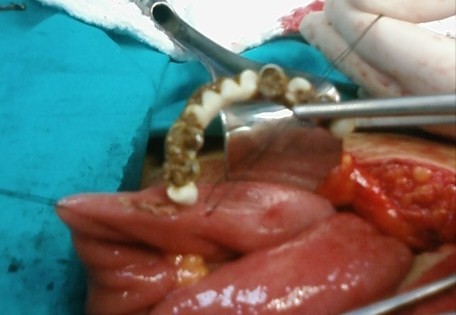
**Extraction of dental denture via ileotomy**.

Because of the persistence of denture at the same region and increase in her complaints we decided to perform surgical exploration. The edema and distention of intestines and hyperemia at the distal ileum were observed. The denture was palpated at entrance of ileocecal valve (Figure 4). The denture was carried backward by gentle maneuver to 20 cm away from ileocecal valve and it was extracted by performing ileotomy (Figure 5). It was a partial denture consisting of eleven units. It has no fixative parts, patient was using a special glue for fixation of denture. The patient's hospital stay was uneventful and discharged at third day postoperatively.

## Discussion

Aspirated or swallowed partial dentures can present a diagnostic challenge. Accidental swallowing is much more common in pediatric group and rare in adults especially reported at with learning and mental health disorders [4,5].

The most likely presenting symptom after swallowing of a denture is dysphasia, with other complaints related to how far the denture has progressed and time since swallowing. Thus further reports may also be anticipated of sore throat, choking sensation, retrosternal pain, sweating and a raised temperature and coughing up blood. Early diagnosis and treatment will avoid the edematous reaction and mucosal infection and necrosis that heighten the risk of rigid oesophagoscopy [6].

Reported late complications of the undiagnosed swallowed denture include extraluminal migration from the esophagus causing either a diverticulum or perforation (once a perforation has occurred, further severe squeal may be anticipated, e.g. tracheo-oesophageal fistula, the need to resect 18 cm of ileum, enterocolonic fistula and sigmoid colon perforation [7-11]. Poly (methylmethacrylate), the plastic from which most dentures are made, is radiolucent. Porcelain teeth produce light shadows on a plain radiograph but it is the metal parts attaching the teeth to the denture base that make them readily visible like in our case. Endoscopy may provide alternative way for extraction of foreign bodies of gastrointestinal tract. In case of failure of the foreign body to progress beyond ileocecal valve colonoscopic extraction may be indicated [12,13]. Ileocecal region is the most frequent site of perforation especially when the object has sharp edges.

We have followed physical examination and radiological findings of this patient for three days. Because of persistence of foreign body image at the same localization and increase of patient's symptoms we have performed surgical exploration to extract the partial denture via enterotomy.

In conclusion, medical personnel, especially those called upon to manage emergencies, should likewise be aware of the multiple hazards. They need to know that certain people will be unable to perceive or report the disappearance of a denture; therefore they should be alert to the possibility of swallowing or inhalation. The follow up of patients must be under the supervision of the same clinician team.

## Consent

Written informed consent was obtained from the patient for publication of this case report and accompanying images. A copy of the written consent is available for review by the Editor-in-Chief of this journal.

## Competing interests

The authors declare that they have no competing interests.

## Authors' contributions

YT collected and interpreted the patient data and performed the surgery with SE and EKO. SE drafted the manuscript. MS and KK helped in collecting of references, figures and drafting of manuscript. MAA critically revised the manuscript. All authors read and approved the final manuscript.
